# Legionella-Induced Rhabdomyolysis and Acute Kidney Injury: A Case Report

**DOI:** 10.7759/cureus.62066

**Published:** 2024-06-10

**Authors:** Pradeep Kumar Mada, Muhammad H Khan

**Affiliations:** 1 Infectious Diseases, Comanche County Memorial Hospital, Lawton, USA; 2 College of Osteopathic Medicine, Michigan State University, East Lansing, USA

**Keywords:** water-borne disease, fluoroquinolones, creatinine kinase. acute kidney injury, acute rhabdomyolysis, legionella pneumonia

## Abstract

Legionella pneumonia is a severe form of pneumonia caused by the bacterium *Legionella pneumophila*. It often presents with atypical symptoms and can lead to complications such as rhabdomyolysis and acute kidney injury (AKI). Here, we report a case of Legionella pneumonia-induced rhabdomyolysis and AKI in a 32-year-old male. Laboratory investigations revealed elevated creatinine kinase levels and acute kidney injury. Further investigation confirmed Legionella pneumonia. The patient was promptly treated with appropriate antibiotics and supportive care, resulting in clinical improvement and resolution of rhabdomyolysis and AKI. This case underscores the importance of considering Legionella pneumonia as a potential cause of rhabdomyolysis and AKI, especially in patients with atypical pneumonia presentations.

## Introduction

Legionella pneumonia is caused by *Legionella pneumophila*, a gram-negative bacterium commonly found in water sources [1]. It is often associated with outbreaks in buildings with complex water systems such as hospitals and hotels [2]. It is a severe form of pneumonia with an incidence of approximately 1.4 to 1.8 cases per 100,000 persons in the United States with a mortality rate of 10-25%. Legionnaires' disease is a nationally notifiable condition in the United States. Reported cases have been rising since the early 2000s [3]. Legionella pneumonia typically presents with nonspecific symptoms, including fever, cough, and shortness of breath. However, it can also present with atypical symptoms such as gastrointestinal symptoms, confusion, and muscle pain [1]. Rhabdomyolysis, characterized by the breakdown of skeletal muscle fibers, and acute kidney injury (AKI) are rare but serious complications of Legionella pneumonia. Here, we present a case of Legionella pneumonia-induced rhabdomyolysis and AKI.

## Case presentation

A 32-year-old incarcerated male with hypertension, asthma, and obstructive sleep apnea (OSA) presented with shortness of breath for five days. He denied chest pain, cough, abdominal pain, and altered bladder or bowel habits. In the emergency department, the patient was febrile at 101.9F, hypertensive at 151/100 mm of hg, tachycardia at 139, tachypnea at 31, and oxygen saturation was 80% on room air. Due to worsening respiratory distress was placed on bi-level positive airway pressure (BiPAP), and subsequently intubated and mechanically ventilated. Labs showed leukocytosis of 16,980, metabolic acidosis, acute kidney injury (AKI), elevated transaminases, rhabdomyolysis, and lactic acid of 4.5 (Table 1). 

**Table 1:** Complete blood count and comprehensive metabolic panel on admission GFR, glomerular filtration rate; HB, hemoglobin; MCH, mean corpuscular hemoglobin; MCHC, mean corpuscular hemoglobin concentration; MCV, mean corpuscular volume; MPV, mean platelet volume; RDW, red cell distribution width; CKD-EPI: Chronic Kidney Disease Epidemiology Collaboration

Parameter	Result	Reference range	Unit
White blood cell count	16.98	4.40-11.00	10x3/mm^3^
Red blood cell count	5.37	4.50-5.90	10x6/mm^3^
Hb	14.2	13.2-16.5	g/dL
Hematocrit	44	39-49	%
MCV	82	80-94	fL
Platelets	205	130-440	10x3/mm^3^
MPV	12.7	7.2-11.1	fL
Neutrophil%	88	40-74	%
Eosinophil%	0.3	0.0-7.0	%
Basophil%	0.6	0.0-1.5	%
Sodium	131	135-145	mmol/L
Potassium	4.0	3.5-5.0	mmol/L
Chloride	95	96-110	mmol/L
Bicarbonate	17	21-31	mmol/L
Glucose	171	80-100	mg/dL
Blood urea nitrogen	35.0	6.0-21.0	mg/dL
Creatinine	2.96	0.6-1.4	mg/dL
Calcium	8.8	8.8-11.1	mg/dL
Total protein	7.2	5.9-8.4	g/dL
Albumin	3.1	3.2-5.2	g/dL
Bilirubin total	1.32	0.00-1.20	mg/dL
Lactic acid	11.5	0.5-2.0	mmol/L
Alkaline phosphatase	85	41-133	u/L
Aspartate aminotransferase	512	7-39	u/L
Alanine aminotransferase	186	2-54	u/L
GFR CKD-EPI	28	60-180	mL/min/1.73m^2^

Infectious workups, including nasal swabs for severe acute respiratory syndrome coronavirus 2 (SARS-CoV-2), flu B, and respiratory syncytial virus (RSV) were negative (Table 2). Sputum culture showed rare growth of typical respiratory flora. Serum Fungitell and galactomannan were negative.

**Table 2:** Infectious workup PCR, polymerase chain reaction; RSV, respiratory syncytial virus; SARS-CoV-2: severe acute respiratory syndrome coronavirus 2

Parameter	Result
Influenza A by PCR	Negative
Influenzae B by PCR	Negative
RSV by PCR	Negative
SARS-CoV-2 by PCR	Negative
Strep Pneumoniae Antigen	Not Detected
HIV 1-2 Antibody/Antigen	Nonreactive
Hepatitis C Antibody	Nonreactive
Hepatitis A Antibody IGM	Nonreactive
Hepatitis B Core Antibody (IgM)	Nonreactive
Hepatitis B Surface Antigen	Nonreactive
Legionella Antigen Urine	Detected

**Figure 1:**
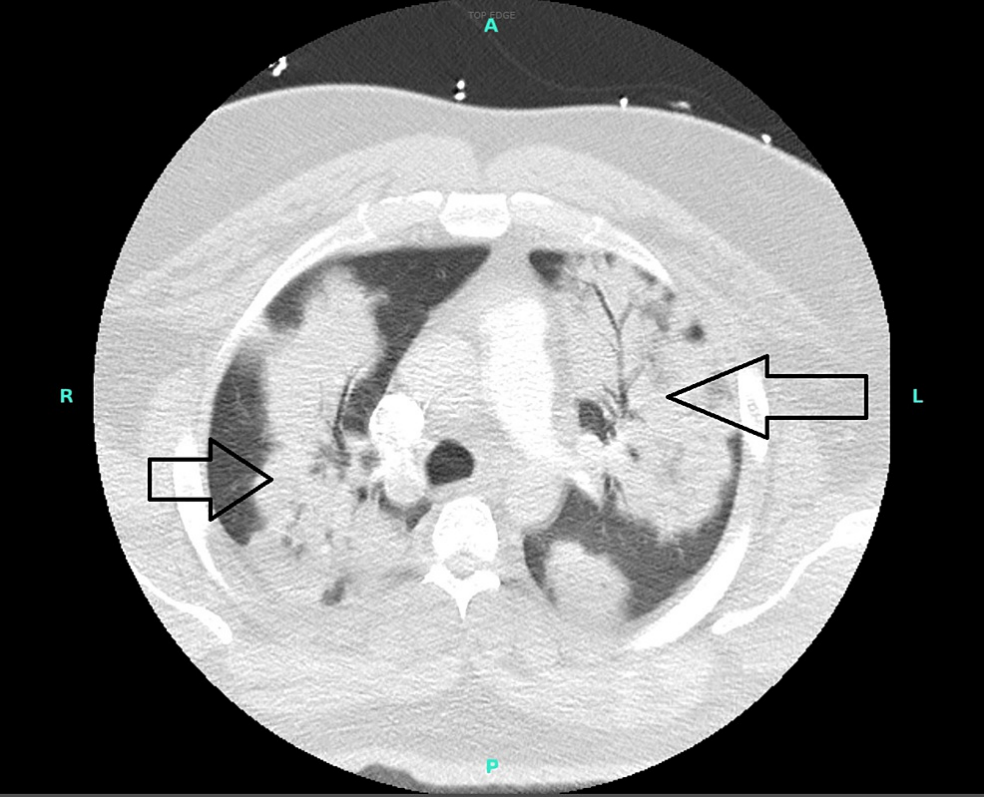
Bilateral multifocal pneumonia (black arrows)

Chest X-ray and contrast-enhanced computed tomography scan of the chest reported multifocal pneumonia (Figure 1).

He was initially started on intravenous vancomycin and meropenem. Urine legionella antigen was positive. Antibiotics were narrowed to levofloxacin, and he completed a two-week course with significant improvement in creatinine kinase (CK) and renal function (Table 3). He was discharged in stable condition.

**Table 3:** CK and creatinine trend CK, creatinine kinase

Day of admission	CK level u/L	Procalcitonin ng/mL	Creatinine mg/dL
Day 1	20601 H	>100.00 HP	2.96
Day 2	28212 H	>100.00 HP	4.06
Day 3	28764 H	>100.00 HP	4.67
Day 4	26212 H	>100.00 HP	4.15
Day 5	12315 H	72.57 HP	3.46
Day 6	10856 H	16.08 HP	2.1
Day 7	7855 H	0.09 H	1.6

## Discussion

Our case highlights the importance of considering Legionella pneumonia in the differential diagnosis of patients presenting with rhabdomyolysis and AKI. Legionnaires’ disease is caused by bacteria belonging to the Legionella genus, and it is an important cause of community and hospital-acquired pneumonia. Transmission occurs via inhalation of aerosols or water containing Legionella species [1]. Legionella is usually found in aquatic environments and multiply rapidly in cases of water stagnation and warm temperatures. Furthermore, locations where the water system has multiple outlets or long pipework are associated with Legionnaires’ disease [2]. Considering the patient's current living situation in a prison, a location with multiple water outlets, we suspect he may have come in contact with the bacteria from the washbasin in his cell or a common area. Subsequent deterioration and disease progression after coming in contact with the bacteria may have been due to his preexisting asthma [4].

While Legionnaires’ disease is known to cause pneumonia, it can also manifest with the involvement of the muscles and kidneys, as was observed in our case. The mechanism of action of rhabdomyolysis secondary to Legionella remains elusive but there have been some mechanisms that have been proposed, including direct invasion of the muscle by the bacteria and release of endotoxins in the bloodstream that lead to subsequent muscle damage [5]. Muscle breakdown leads to the buildup of myoglobin in the kidney tubules, which can eventually lead to AKI. Normally, AKI occurs in septic shock or hypotension, which our patient did not present with [6]. Therefore, it is likely he developed AKI secondary to rhabdomyolysis secondary to Legionella pneumonia, which typically requires aggressive fluid administration [7].

We used urinary antigen detection to diagnose Legionnaires’ disease quickly. While we successfully detected the bacteria, there is a possibility of missing as much as 40% of cases, as the urine antigen test is limited to detecting a single strain [8,9]. As an alternative, PCR, which can detect all Legionella strains, has been implemented in a few clinical laboratories but its wide-scale use is being questioned due to varying sensitivity and specificity [9]. In practice, multiple diagnostic tools, including imaging and cultures, may be necessary to avoid missing the diagnosis.

The primary goal of antimicrobial therapy in Legionnaires’ disease is to select an antibiotic that can accumulate in large concentrations within the cells [10]. These include macrolides, quinolones, ketolides, tetracyclines, and rifampin, with quinolones having the highest intracellular activity against Legionella [11]. In cases of severe disease, certain fluoroquinolones, like levofloxacin, are safe and effective for treatment [12]. A meta-analysis showed no difference in the effectiveness of fluoroquinolones versus macrolides in reducing mortality among patients with Legionella pneumonia [13]. In our case, we found success in using levofloxacin for two weeks after the patient was stabilized and discharged.

## Conclusions

In conclusion, Legionella pneumonia-induced rhabdomyolysis and AKI are rare but potentially life-threatening complications that should be considered in patients presenting with atypical pneumonia symptoms. Prompt recognition and treatment are essential to prevent further complications and improve outcomes. Clinicians should maintain a high index of suspicion for Legionella pneumonia in patients presenting with rhabdomyolysis and AKI, especially in the context of outbreaks or clusters of pneumonia cases.
